# Supplementary material for: CRISPRi chemical genetics and comparative genomics identify genes mediating drug potency in Mycobacterium tuberculosis
Source: Nat Microbiol. 2022 May 30;7(6):766–79. doi: 10.1038/s41564-022-01130-y (PMC9159947; doi:10.1038/s41564-022-01130-y)
Supplement: Supplementary file 1 — Supplementary Method, References and Tables 1–4. [file 41564_2022_1130_MOESM1_ESM.pdf]

nature portfolio

https://doi.org/10.1038/s41564-022-01130-y

Supplementary information

CRISPRi chemical genetics and comparative genomics identify genes mediating drug potency in *Mycobacterium tuberculosis*

In the format provided by the authors and unedited

Supplementary Information

To

CRISPRi chemical genetics and comparative genomics identify genes mediating drug potency in *Mycobacterium tuberculosis*

Shuqi Li[1*], Nicholas C. Poulton[1*], Jesseon S. Chang[1], Zachary A. Azadian[1], Michael A. DeJesus[1], Nadine Ruecker[2], Matthew D. Zimmerman[3], Kathryn A. Eckartt[1], Barbara Bosch[1], Curtis A. Engelhart[2], Daniel F. Sullivan[2], Martin Gengenbacher[3,4], Véronique A. Dartois[3,4], Dirk Schnappinger[2], Jeremy M. Rock[1]

1: Laboratory of Host-Pathogen Biology, The Rockefeller University, New York, New York, United States of America

2: Department of Microbiology and Immunology, Weill Cornell Medicine, New York, New York, United States of America

3: Center for Discovery and Innovation, Hackensack Meridian Health, Nutley, New Jersey, USA

4: Hackensack Meridian School of Medicine, Hackensack Meridian Health, Nutley, New Jersey, USA

*: co-first authors

Address correspondence to: rock@rockefeller.edu

This Supplementary Information file contains the following:

• Supplementary Methods Page 2-4
• Reference for Supplementary Methods Page 4
• Supplementary Tables 1-4 Page 5-11

Supplementary Methods

Construction of the Δ*whiB7* and complemented Mtb strains

The Mtb Δ*whiB7* strain was constructed by allelic exchange using a RecET-mediated recombineering approach as previously described[1]. Deletion of *whiB7* was confirmed by PCR and whole-genome sequencing (BGI). The Δ*whiB7* strain was complemented by reintroducing a wild-type copy of *whiB7* expressed from its native promoter integrated at *attL5*. Plasmid sequences and maps can be found in **Supplementary Table 2**.

Antimicrobial compounds

All compounds used in this study **(Supplementary Table 4)** were purchased from commercial manufacturers with the exception of GSK3011724A, which was synthesized at the Memorial Sloan Kettering Organic Synthesis core as described in[2].

NGS data processing, analysis, and hit calling

Sequencing counts were obtained in the manner described by[3]. Counts were normalized for sequencing depth and an sgRNA limit of detection (LOD) cut-off was set at 100 counts in the DMSO condition. Only sgRNAs that made the LOD cut-off (i.e. counts > 100) were analyzed further. Replicate screens were quality controlled to ensure that the Pearson correlation was >0.95 for both the non-targeting sgRNA sets and essential-gene targeting sgRNA[3] sets between each replicate screen. sgRNA counts were analyzed using MAGeCK (version 0.5.9.2) in python (version 2.7.16)[4] comparing each drug treatment condition to the matched vehicle control (DMSO) sample. Gene-level log2 fold change (L2FC) was calculated using the 'alphamedian' approach specified with the 'gene-lfc-method' parameter, which estimates the gene-level L2FC as the median of sgRNAs that are ranked above the default cut off in the Robust Rank Aggregation used by MAGeCK.

Negative control sgRNAs were used to calculate the null distribution and to normalize counts using the '--control-sgrna' and '–normalization control' parameters, respectively. MAGeCK gene summary output results can be found in **Source Data 1.**

Unless otherwise specified, a gene was determined to be a hit in a given condition if it had a false discovery rate (FDR) < 0.01 and a log2 fold change (L2FC) < -1 in negative selection or L2FC > 1 in positive selection.

Clustered heatmap

L2FC values from the MAGeCK output (**Source Data 1**) were used for the generation of a clustered heatmap. Genes were clustered based on Euclidean distance using the Ward clustering criterion. Only genes that hit (FDR < 0.01; n=676 genes) in two or more conditions are included in the heatmap. A condition is defined as any single drug concentration screen (e.g. rifampicin "High"). A gene's L2FC was only represented in the heatmap if the FDR was below 0.01 in the specific treatment condition; genes not meeting this significance threshold are shown as white in the relevant treatment condition. Treatment conditions were clustered based on Pearson correlation using the Ward criterion. Clustering was done using the package hclust and the heatmap was generated using the heatmap.2 function from the package gplots (R version 4.0.5).

RNA-seq cDNA library construction and deep sequencing

Triplicate cultures were grown to mid-log phase in 7H9 and diluted back to OD_{600}=0.2 in 7H9 in the presence or the absence of ATc (100 ng/mL). Cultures were incubated for 48 hours, after which total RNA was extracted as described in "*Total RNA extraction and qRT-PCR*." Following RNA cleanup (Zymo Research R1017), 2 μg total RNA for each sample was depleted for rRNA using a Ribominus Transcriptome Isolation Kit (Invitrogen K1550-

03). Following rRNA depletion, RNA was concentrated using an RNA Clean and Concentration-5 kit (Zymo Research R1013). RNA quality was then confirmed by Bioanlayzer (Agilent RNA 6000 Pico 5067-1513).

We used the NEB Next Ultra II Directional RNA Library Prep Kit (NEB E7760 and E7765) to prepare cDNA libraries following manufacturer's instructions. Briefly, 150 ng of rRNA-depleted RNA was subjected to fragmentation by incubating samples at 94°C for 20 min, followed by first strand cDNA synthesis (10 minutes at 25°C, 50 minutes at 42°C, 15 minutes at 70°C, hold at 4°C). Second-strand synthesis was performed at 16°C for 1.5 hours. DNA purification was performed with AMPure XP beads (Beckman Coulter A63881). End repair was performed for 30 minutes at 20°C, followed by 30 minutes at 65°C. Repaired dsDNA was adaptor ligated (15 minutes at 37°C) and purified with AMPure XP beads. Eluted DNA was amplified by PCR using NGS primers supplied with the kit (NEBNext Multiplex Oligos for Illumina, Index Primers Set 1 and 2, E7335S, E7500S) for 12 cycles of amplification. Amplicons were purified with AMPure XP beads, quantified by Qubit dsDNA HS Assay kit (TheromoFisher Scientific Q32851), and quality controlled by BioAnalzer (Agilent DNA 1000 5067-1504). RNAseq libraries were sequenced on an Illumina NextSeq 500 (mid-output, 75 bp paired-end reads).

Processing and analysis of RNA-seq data

Raw FASTQ files were aligned to the H37Rv genome (NC_018143.2) using Rsubread (version 2.0.1)[5] with default settings. Transcript abundances were calculated by processing the resulting BAM files with the summarizeOverlaps function of the R package GenomicAlignments (version 1.22.1)[6]. Overlaps were calculated in the "Union" mode, ensuring reads were counted only if they overlap a portion of a single gene/feature. 16S, 23S, and 5S rRNA features (RVBD6018, 6019, and 6020, respectively) were manually removed from the count data to prevent confounding downstream differential gene expression analysis. Differential expression analysis was conducting using DESeq2 (version 1.30.1)[7] with default parameters.

Cell wall permeability assay

Cell envelope permeability was determined using the ethidium bromide (EtBr) uptake assay as previously described[8]. Briefly, mid-log-phase Mtb cultures were washed once in PBS + 0.05% Tween-80 and adjusted to OD_{600}=0.8 in PBS supplemented with 0.4% glucose. 100 µL of bacterial suspension was added to a black 96-well clear-bottomed plate (Costar). After this, 100 µL of 2 µg/mL EtBr in PBS supplemented with 0.4% glucose was added to each well. EtBr fluorescence was measured (excitation: 530 nm/emission: 590 nm) at 1 min intervals over a course of 60 min. Experiments were performed in technical triplicate.

A similar assay was performed to determine envelope permeability to a fluorescent vancomycin analogue, except that: (1) the bacterial suspension was adjusted at OD_{600}=0.4 in PBS supplemented with 0.4% glucose; (2) cells were incubated with 2 µg/mL BODIPY FL Vancomycin (Thermo Scientific V34850); (3) 200 µL sample aliquots were taken at different time points, washed twice with PBS, resuspended in 200 µL PBS; and (4) fluorescence was measured (excitation: 485 nm/emission: 538 nm) and normalized to the OD_{600} of the final bacterial suspension.

Whole genome sequencing data aggregation, alignment, SNP calling and annotation

FASTQ data were downloaded from NCBI using the SRA Toolkit (version 2.9.6). A list of accession numbers of all analyzed FASTQ files is provided in **Source Data 4**. FASTQ reads were aligned to the H37Rv genome (NC_018143.2) and SNPs were called and annotated using Snippy[9] (version 3.2-dev) using default parameters (minimum mapping quality of 60 in BWA, SAMtools base quality threshold of 20, minimum coverage of 10, minimum proportion of reads that differ from reference of 0.9). Mapping quality and coverage was further assessed using QualiMap with the default parameters (version 2.2.2-dev)[10] . Samples with a mean coverage < 30, mean mapping quality <= 45, or GC content <= 50% or >= 70% were excluded. Spoligotypes were assigned using SpoTyping (version 2.1)[11]. Drug resistance conferring SNPs were annotated using Mykrobe v0.9.0[12].

Phylogenetic trees were built using FastTree (version 2.1.11 SSE3)[13]. A list of SNPs in essential genes was concatenated to build phylogenetic trees. Indels, drug resistance-conferring SNPs, and SNPs in repetitive regions of the genome (PE/PPE genes, transposases and prophage genes) were excluded. Tree visualization was performed in iTol (https://itol.embl.de/).

Sublineage identification

Mtb sublineages were assigned to each sample using a set of lineage identifying SNPs. Lineage identifying SNPs from[14,15] were combined and then reduced to a subset of synonymous SNPs occurring in essential genes. For each sample, the percentage of lineage identifying SNPs present was calculated for each possible sublineage. A threshold of 67% was set as the minimum percentage of sublineage identifying SNPs required in order to define a sublineage. For each sample, all sublineages meeting this threshold were then evaluated to determine if they formed a continuous line of descent from the highest sublineage to the lowest (e.g. lineage1 → lineage1.2 → lineage1.2.1 → lineage1.2.1.1). Samples with a continuous line of descent were assigned the most specific sublineage (e.g. lineage1.2.1.1). If the set of sublineages included other sublineages that did not fit within the line of descent, the sublineage call was marked as “not confident” and considered as an undetermined sublineage.

References for Supplementary Methods

1. Murphy, K. C., Papavinasasundaram, K. & Sassetti, C. M. Mycobacterial recombineering. *Methods Mol. Biol.* **1285**, 177–99 (2015).
2. Kumar, P. *et al.* Synergistic Lethality of a Binary Inhibitor of *Mycobacterium tuberculosis* KasA. *MBio* **9**, (2018).
3. Bosch, B. *et al.* Genome-wide gene expression tuning reveals diverse vulnerabilities of *M. tuberculosis*. *Cell* **184(17)**, 4579–4592 (2021).
4. Li, W. *et al.* MAGeCK enables robust identification of essential genes from genome-scale CRISPR/Cas9 knockout screens. *Genome Biol.* **15**, 554 (2014).
5. Liao, Y., Smyth, G. K. & Shi, W. The R package Rsubread is easier, faster, cheaper and better for alignment and quantification of RNA sequencing reads. *Nucleic Acids Res.* **47**, (2019).
6. Lawrence, M. *et al.* Software for Computing and Annotating Genomic Ranges. *PLoS Comput. Biol.* **9**, 1–10 (2013).
7. Love, M. I., Huber, W. & Anders, S. Moderated estimation of fold change and dispersion for RNA-seq data with DESeq2. *Genome Biol.* **15**, 1–21 (2014).
8. Xu, W. *et al.* Chemical Genetic Interaction Profiling Of Intrinsic Antibiotic Resistance in *Mycobacterium tuberculosis*. *Antimicrob. Chemother* **61**, 1–15 (2017).
9. Bush, S. J. *et al.* Genomic diversity affects the accuracy of bacterial single-nucleotide polymorphism-calling pipelines. *Gigascience* **9**, 1–21 (2020).
10. Okonechnikov, K., Conesa, A. & García-Alcalde, F. Qualimap 2: Advanced multi-sample quality control for high-throughput sequencing data. *Bioinformatics* **32**, 292–294 (2016).
11. Xia, E., Teo, Y. Y. & Ong, R. T. H. SpoTyping: Fast and accurate in silico *Mycobacterium* spoligotyping from sequence reads. *Genome Med.* **8**, 19 (2016).
12. Hunt, M. *et al.* Antibiotic resistance prediction for *Mycobacterium tuberculosis* from genome sequence data with Mykrobe. *Wellcome open Res.* **4**, 191 (2019).
13. Price, M. N., Dehal, P. S. & Arkin, A. P. FastTree 2 - Approximately maximum-likelihood trees for large alignments. *PLoS One* **5**, (2010).
14. Coll, F. *et al.* A robust SNP barcode for typing *Mycobacterium tuberculosis* complex strains. *Nat. Commun.* **5**, (2014).
15. Palittapongarnpim, P. *et al.* Evidence for Host-Bacterial Co-evolution via Genome Sequence Analysis of 480 Thai *Mycobacterium tuberculosis* Lineage 1 Isolates. *Sci. Rep.* **8**, 1–14 (2018).

Supplementary Tables

Supplementary Table 1: Drugs Profiled and their Target Processes

Drug	Abbreviation	Class	Target Process
Bedaquiline	BDQ	Diarylquinoline	ATP synthesis
Clarithromycin	CLR	Macrolide	Translation
Ethambutol	EMB	Ethylenediamine	Arabinogalactan biosynthesis
Isoniazid	INH	Pyridine	Mycolic acid biosynthesis
Levofloxacin	LVX	Fluoroquinolone	DNA replication
Linezolid	LZD	Oxazolidinone	Translation
Rifampicin	RIF	Rifamycin	Transcription
Streptomycin	STR	Aminoglycoside	Translation
Vancomycin	VAN	Glycopeptide	Peptidoglycan biosynthesis

Supplemental Table 2: List of plasmids and primers used in this work

Plasmids used in this work

Fig. used	Plasmid Name	Plasmid genotype	Plasmid description	Plasmid Map	Resistance marker
2,3,4,5, S4, S5, S6, S7, S8,S9	pIRL58 (Addgene #166886)	Ptet(gB73)-Sth1 dCas9 Ptet(gB52)-Sth1 sgRNA P(gB37)-TetRCO(tetON) L5 attP only v1::Kan	Sth1 dCas9 CRISPRi plasmid optimized for use in *M. tuberculosis*. Sth1 dCas9 and the sgRNA are induced in the presence of ATc. This plasmid lacks the full L5 integrase and must be co-transformed pIRL19	https://benchling.com/s/seq-Gv9EfPn1vM7OMu6HPyU3	Kanamycin
2,3,4,5, S4, S5, S6, S7, S8,S9	pIRL19 (Addgene #163634)	Pmop-L5 Int AmpR (suicide plasmid)	The L5 phage Int protein is expressed from the mycobacterial optimized promoter (MOP). This backbone is non-replicating and non-integrating in mycobacteria.	https://benchling.com/s/seq-BAF0JelwBQN6D2dw5alf	Ampicillin
2, S5, S8	pIRL60	pDE43-MCZtq26 Tweety attP::Zeo (empty vector)	Tweety::Zeo integration backbone. Contains the Tweety Int with a zeocin resistance cassette.	https://benchling.com/s/seq-s6M31jHfAUSXmCl90JQc	Chloramphenicol/ Zeocin
2, S5	pIRL140	pIRL60-PmtrA-mtrA Tweety attP::Zeo	CRISPRi resistant allele of the mtrA ORF under the expression of the endogenous mtrA promoter (300 bp upstream of the mtrA translational start site). Cloned by Gibson assembly into EcoRV and XbaI-digested pIRL60.	https://benchling.com/s/seq-T2YS6JwBvXhdDigP54Wp	Chloramphenicol/ Zeocin
2, S5	pIRL141	pIRL60-PmtrA-mtrB Tweety attP::Zeo	CRISPRi resistant allele of the mtrB ORF under the expression of the endogenous mtrA promoter (300 bp upstream of the mtrA translational start site). Cloned by Gibson assembly into EcoRV and XbaI-digested pIRL60.	https://benchling.com/s/seq-BaNN2970G1ukBaFnqZ3b	Chloramphenicol/ Zeocin
2, S5	pIRL142	pIRL60-Phsp60-lpqB Tweety attP::Zeo	CRISPRi resistant allele of the lpqB ORF under the expression of the hsp60 promoter. Cloned by Gibson assembly into EcoRV and XbaI-digested pIRL60.	https://benchling.com/s/seq-n98842nj4uVFdah5COhw	Chloramphenicol/ Zeocin
4, 5, S7	pIRL133	pIRL133 Giles attP::Zeo (empty vector)	Vector used for bacA and ettA experiments. Integrating vector containing the Giles attP site and expressing a zeocin resistnace casstte. Must be co-transformed with pIRL40. Contains barcode random barcode sequence AAATAAAAACCACTCTCC	https://benchling.com/s/seq-m7KsnT49GbAKHZOruvjn	Zeocin
4, 5, S7	pIRL40	Puv15-Giles Int (suicide plasmid)	The Giles phage Int protein is expressed from the Puv15 promoter. This backbone is non-replicating and non-integrating in mycobacteria.	https://benchling.com/s/seq-2PVXvHq0CfNPVacwz1Ku	Ampicillin
4, S7	pINP411	pIRL133-Phsp60-bacA WT Giles attP::Zeo	pIRL133 backbone expressing the WT bacA allele under the hsp60 promoter. Contains random barcode sequence TCGGGAATTCTCACGCGT	https://benchling.com/s/seq-xm930WSRuZg57YePOv6V	Zeocin
4	pINP412	pIRL133-Phsp60-bacA Trp153Ser Giles attP::Zeo	pIRL133 backbone expressing the Trp153Ser bacA allele under the hsp60 promoter. Contains random barcode sequence ATCAGTGTTTCATACAAG	https://benchling.com/s/seq-xS0HjAjiI3meB6dNLvRZ	Zeocin
4	pINP413	pIRL133-Phsp60-bacA Tyr288Stop Giles attP::Zeo	pIRL133 backbone expressing the Tyr288Stop bacA allele under the hsp60 promoter. Contains random barcode sequence GCCGTTTGGGACTCGTCT	https://benchling.com/s/seq-wc5ZxwPFHxRtKA64bRKt	Zeocin
S7	pINP415	pIRL133-Phsp60-bacA Asp546Ala Giles attP::Zeo	pIRL133 backbone expressing the Asp546Ala bacA allele under the hsp60 promoter. Contains random barcode sequence ATACTCAAGTTTATATAT	https://benchling.com/s/seq-vLyBVRz5sgy98D3Kevt6	Zeocin

4	pINP416	pIRL133-Phsp60-bacA Leu551fs Giles attP::Zeo	pIRL133 backbone expressing the Leu551fs bacA allele under the hsp60 promoter. Contains random barcode sequence GGGAGTCTGTCTCTACCA	https://benchling.com/s/seq-7qlml27CxTswBCArMKaw	Zeocin
4	pINP417	pIRL133-Phsp60-bacA Gly554fs Giles attP::Zeo	pIRL133 backbone expressing the Gly554fs bacA allele under the hsp60 promoter. Contains random barcode sequence TTGGTACCCCGTTATAGT	https://benchling.com/s/seq-PjFZhXAUmUCuo0V1kSwi	Zeocin
S7	pINP422	pIRL133-Phsp60-bacA Val118Ala Giles attP::Zeo	pIRL133 backbone expressing the Val118Ala bacA allele under the hsp60 promoter. Contains random barcode sequence TTGCTCATATTCGCGGTA	https://benchling.com/s/seq-2gsmPyD9adeI4O452V1c	Zeocin
S7	pINP370	Ptet(gB52)-bacA L5 attP: Kan	L5 integrating plasmid constitutively expressing the Mtb bacA allele under the synthetic Ptet(gB52) promoter in the absence of a Tet repressor	https://benchling.com/s/seq-WDve9cLzU2bhPwCEhNCM	Kanamycin
S7	pINP371	Ptet(gB52)-Empty L5 attP: Kan	L5 integrating plasmid with the synthetic Ptet(gB52) promoter and no downstream gene. As with pINP370 this plasmid lacks a Tet repressor	https://benchling.com/s/seq-84uPVQxdtoa9RgPuli6N	Kanamycin
5, S7	pIRL134	pIRL133-PettA-ettA WT Giles attP::Zeo	pIRL133 backbone expressing a CRISPRi resistant WT allele of ettA under its endogenous promoter. Contains random barcode sequence TCGGGAATTCTCACGCGT	https://benchling.com/s/seq-kKKtvttIAOgThvBiZoD7	Zeocin
S7	pIRL135	pIRL133-PettA-ettA Pro39Ser Giles attP::Zeo	pIRL133 backbone expressing a CRISPRi resistant Pro39Ser allele of ettA under its endogenous promoter. Contains random barcode sequence ATCAGTGTTTCATACAAG	https://benchling.com/s/seq-m7KsnT49GbAKHZOruvjn	Zeocin
5, S7	pIRL136	pIRL133-PettA-ettA Gly41Glu Giles attP::Zeo	pIRL133 backbone expressing a CRISPRi resistant Gly41Glu allele of ettA under its endogenous promoter. Contains random barcode sequence GCCGTTTGGGACTCGTCT	https://benchling.com/s/seq-2YfWjMdXzrg5u8DjRT3U	Zeocin
5, S7	pIRL137	pIRL133-PettA-ettA Trp135Gly Giles attP::Zeo	pIRL133 backbone expressing a CRISPRi resistant Trp135Gly allele of ettA under its endogenous promoter. Contains random barcode sequence ATACTCAAGTTTATATAT	https://benchling.com/s/seq-HMeozzDPogc92G7zvBbR	Zeocin
5, S7	pIRL138	pIRL133-PettA-ettA Val426Ala Giles attP::Zeo	pIRL133 backbone expressing a CRISPRi resistant Val426Ala allele of ettA under its endogenous promoter. Contains random barcode sequence TTGGTACCCCGTTATAGT	https://benchling.com/s/seq-poxjOoLz4VkRb2pyW8Nc	Zeocin
5, S7	pIRL139	pIRL133-PettA-ettA Val426Met Giles attP::Zeo	pIRL133 backbone expressing a CRISPRi resistant Val426Met allele of ettA under its endogenous promoter. Contains random barcode sequence GTCGGCTAGCAATATTCT	https://benchling.com/s/seq-Cet0qyRkZVDzWjqk4Fu5	Zeocin
S8	pIRL145	pBR322-whiB7::Amp	Backbone: pBR322. Cloned whiB7+5'UTR region, as PCR template for generating whiB7 KO mutant in Mtb.Cloned by Gibson assembly into ClaI and HindIII-digested pIRL39.	https://benchling.com/s/seq-8HUz8kLA4QtU3gEkTirl	Ampicillin
S8	pIRL146	pIRL60-PwhiB7-whiB7-Gly64delG(fs) Tweety attP::Zeo	whiB7-Gly64delG mutant ORF under the expression of the endogenous whiB7 promoter (500 bp upstream of the whiB7 translational start site including the 5'UTR region). Cloned by Gibson assembly into EcoRV and XbaI-digested pIRL60.	https://benchling.com/s/seq-EYD2RXXj3Zk3Rojw5ruG	Chloramphenicol/ Zeocin
S10	pIRL165	pIRL133-PmmpS5/L5-mmpS5/L5 Giles attP::Zeo	pIRL133 backbone expressing the mmpS5/L5 operon driven by the endogenous promoter. Contains random barcode sequence TGCCCACCGTGCCTCGTT	https://benchling.com/s/seq-M4TANSLiyI5qlcL88k0v?m=slm-lCd3bvbRgpMtgMaMM3Er	Zeocin
S10	pIRL166	pIRL133-Phsp60-mmpS5/L5 Giles attP::Zeo	pIRL133 backbone expressing the mmpS5/L5 operon driven by the hsp60 promoter. Contains random barcode sequence TGCCCACCGTGCCTCGTT	https://benchling.com/s/seq-6RKnURqDTWuIUg93EGfF?m=slm-Gumk1xjfRfkxlrx1TbHI	Zeocin

sgRNAs used in this work

Fig. used	sgRNA ID	Gene targeted	Gene name	sgRNA targeting sequence (5'–3')	PAM (5'–3')
2,3,4,5,6 S4, S5, S6, S7, S8	Non-Targeting control	NA	NA	GCATCCGGAGCCCGTCCGTTAA	NA
S4	*ccdA* sgRNA PAM 15	*rv0527*	*ccdA*	ATCAGCACGCCTCCGACCCGCT	GCAGCAG
S4	*kasA* sgRNA PAM 22	*rv2245*	*kasA*	ATGTCGTGCTTCAGTAACGCCCG	AAGGCAA
S4	*mmpL3* sgRNA PAM 15	*rv0206c*	*mmpL3*	AACATCCGCACCACGGTCGCGT	CCAGCAG
S4	*embB* sgRNA PAM 8	*rv3795*	*embB*	GACAGCGAACCGTCGACGGTGG	GCAGGAT
S4	*ripA* sgRNA PAM 10	*rv1477*	*ripA*	GCTGCAGGTTGGCCATCACCGCT	TGGGAAC
2, S5	*mtrA* sgRNA PAM 4	*rv3246c*	*mtrA*	GGTGAGCATCACGATCGGAACA	CCGGAAT
2, S5	*mtrB* sgRNA PAM 2	*rv3245c*	*mtrB*	GTCCCGATGATCAGGGCCGGCCC	GGAGAAA
2, S5	*lpqB* sgRNA PAM 1	*rv3244c*	*lpqB*	ACCCCCACATCCGAGAGCGAGC	CGAGAAT
3, S6	*smpB* sgRNA PAM 15	*rv3100c*	*smpB*	GTGTCGATCTGGCGGCGATGCA	ACAGCAG
3, S6	*rv1473* sgRNA PAM 1	*rv1473*	*rv1473*	GCATAGGGTTCGACCTCCCCCG	CCAGAAT
S6	*clpP2* sgRNA PAM 16	*rv2460c*	*clpP2*	GATGTACATGGTGATATCGCGG	TCGGGAT
5, S8	*ettA* sgRNA PAM 2	*rv2477c*	*ettA*	ACGACACCGATCTTGGCGCCCGG	ATAGAAA
5	*whiB7* promoter sgRNA PAM 6	*rv3197A*	*whiB7*	GCCTGTACCGGCAAACGCGCAGG	TCAGAAA
5	*tap* sgRNA PAM 5	*rv1258c*	*tap*	GCCCTCGCGCTGCAACACCAGCC	ACGGAAA
5	*eis* sgRNA PAM 1	*rv2416c*	*eis*	GTCGGGCTACACAGGGTCACAGTC	ACAGAAT

qPCR primers used in this work

Fig. used	Target gene	Gene name	Forward primer (5'-3')	Reverse primer (5'-3')
2, 5, S5	*rv2703*	*sigA*	GTGATTTCGTCTGGGATGAAGA	TACCTTGCCGATCTGTTTGAG
2, S5	*rv3246c*	*mtrA*	CCATCGCCGACGTAGAAAT	AGCAGCACATCACGAGTAAA
2, S5	*rv3245c*	*mtrB*	ACCACGCCGAACACAAA	AAACACCAGCTTCTCCTCAC
2, S5	*rv3244c*	*lpqB*	GTGCTGCGAGCGATACA	GTCACGGGACAGTTGAAGAT
2, S5	*rv1477*	*ripA*	TACTCGGGTTCGCAGTACAA	TTCGGGCCGTAGAAGATGA
2, S5	*rv3810*	*pirG*	AACGACGTGATGCAGGTG	TGCATGATCGACGGCATTAG
2, S5	*rv0040c*	*mtc28*	CATCACACACGGCTACATTGA	CCTCGATGATTGATGACGGAAA
2, S5	*rv1158*	*rv1158*	CCAACGCACCGCAAATC	TGGAAAGGTGGCGGTTATC
2, S5	*rv0312*	*rv0312*	CCATTGACCGGCTTCATCTA	GACGAGTGTGGAACGAAAGA
2, S5	*rv1566*	*rv1566*	CTGTCCTGGTTTACGGTCTG	AACCCGACGGTGTTGATG
2, S5	*rv0129c*	*fbpC*	GTGTTGCTGGACGGAACTAA	GGATGGCACCTGCAGATATT
5	*rv3197a*	*whiB7*	CAGACAAAGATTGCCGGTTT	ACACACAGTGTCTTGGCTAC
5	*rv1988*	*erm*	CCGCCGTACGGGATTTC	CGTTGCGAGAAGCGAATTTAC
5	*rv2725c*	*hflx*	GCTGTGCAGGTTAAGGTTATTG	GCAGCATGTACTCCATCTGAG
5	*rv2416c*	*eis*	TCACGAAGTTGGCGAGTTT	CAGTACGTCCCGATCCATTTC

Other primers used in this work

Fig. used	Legacy Name	Sequence (5'-3')	Notes	Description
3, S6	olNP537	GGTTGGGCAAGAGTTGACCGCG	rplC Amplification Fwd Primer	rplC Amplification Fwd Primer, LZD-resistant Mtb
3, S6	olNP538	TCTGCTCTTGCGCAGCCATCAC	rplC Amplification Rev Primer	rplC Amplification Rev Primer, LZD-resistant Mtb
S10	olNP531	GCATACCAAGGCGTACGAGATAAC	Rrl Amplification Fwd Primer	Rrl Amplification Fwd Primer, CLR-resistant Mtb
S10	olNP532	CTGATCTTGGAGAAGGTTTCCCG	Rrl Amplification Rev Primer	Rrl Amplification Rev Primer, CLR-resistant Mtb
6, S9	olLSQ564	GAGGCCCTTTCGTCTTCAAGCGCCGATC CGGTGCCGGG	whiB7-mtb-500_fwd	whiB7-KO Mtb mutant construction
6, S9	olLSQ565	TGGATCCACTGCCGACACCAAATGCGTTG TTGTCAATCAC	whiB7-mtb-500_rev	whiB7-KO Mtb mutant construction
6, S9	olLSQ566	TGGTGTCGGCAGTGGATCCATAACTTCGT ATAATGTATG	whiB7-mtb-hyg_fwd	whiB7-KO Mtb mutant construction
6, S9	olLSQ567	GACGTCCGCGGGCGCGCCATAACTTCGT ATAG	whiB7-mtb-hyg_rev	whiB7-KO Mtb mutant construction
6, S9	olLSQ568	ATGGCGCGCCCGCGGACGTCCGCGCAA G	whiB7-mtb-500_fwd	whiB7-KO Mtb mutant construction
6, S9	olLSQ569	GTGATAAACTACCGCATTAATGCTGCTGC CCGACGGCC	whiB7-mtb-500_rev	whiB7-KO Mtb mutant construction
6, S9	olLSQ608	CTGATCGGTACGCTGCTCGCCG	whiB7-KO-veri-F	Validation primer to confirm removal of endogenous whiB7 allele
6, S9	olLSQ609	AGTCCATTTGGCGCTCAGCTG	whiB7-KO-veri-R	Validation primer to confirm removal of endogenous whiB7 allele
6, S9	olLSQ457	CCTCTAGGGTCCCCAGCTGGCCGGCATC GGTGCCCGCA	WhiB7+500-gibson-F	Build whiB7 WT allele for complementation
6, S9	olLSQ458	GTGGCAGGGCGGGGCGTAATCTATGCAA CAGCATCCTTGCGCGG	WhiB7+500-gibson-R	Build whiB7 WT allele for complementation
6, S9	olLSQ669	CCTCTAGGGTCCCCAGCTGGCCGGCATC GGTGCCCGCA	pwhiB7-whiB7fs-F1	Build Mtb whiB7-fs(Gly64delG) allele for complementation
6, S9	olNP514	ATCCTATGCCAGCTGGACGC	whiB7 Amplification Fwd Primer	whiB7 Amplification Fwd Primer
6, S9	olNP546	CCCGCAAGCTGGAACAATAC	whiB7 Amplification Rev Primer	whiB7 Amplification Rev Primer

Supplementary Table 3: Additional acquired drug sensitivity candidate genes

Gene	Drug	Mutation	Sublineage	Occurrences	Notes
whiB7	CLR	Leu18fs	lineage2.2.1: 100/6719	100	
whiB7	CLR	Ala60fs	lineage4: 2/814, lineage4.8: 18/3234	20	
hflX	CLR	Glu206fs	lineage4.9: 93/2941	93	
hflX	CLR	Arg237fs	lineage4.9: 12/2941	12	
hflX	CLR	Gln367Stop	lineage4.2.1: 117/666, lineage4.6: 2/187	119	
rv2369c	CLR	Arg57fs	None: 12/3241	12	Possible polar effect with *phoH1*
rv2369c	CLR	Cys58fs	lineage3: 11/3537	11	Possible polar effect with *phoH1*
rv2369c	CLR	Gln72Stop	lineage1.2.1: 2/164, lineage1.2.1.3: 78/435	80	Possible polar effect with *phoH1*
mmpL5	CLR/BDQ	Asp132fs	None: 48/3241, lineage1.2.1: 1/164, lineage2.2.1: 5/6719, lineage4.1.1.3: 2/1178, lineage4.8: 1/3166	57	Also observed in PMID: 32143680, may also confer clofazimine sensitivity
mmpL5	CLR/BDQ	Arg202fs	lineage4: 55/807	55	Also observed in PMID: 32143680, may also confer clofazimine sensitivity
mmpL5	CLR/BDQ	Tyr300Stop	lineage1.1.1: 4/54, lineage1.1.1.1: 75/108, lineage4.8: 1/3166	80	Also observed in PMID: 32143680, may also confer clofazimine sensitivity
mmpL5	CLR/BDQ	Pro498fs	lineage2.2.2: 1/747, lineage4.6: 59/187	60	Also observed in PMID: 32143680, may also confer clofazimine sensitivity
rv3822	BDQ	Leu123fs	None: 40/2223	40	
rv3822	BDQ	Tyr208fs	lineage4: 4/814, lineage4.8: 36/3234	40	

Supplementary Table 4: Antimicrobial compounds used in this study

Antibiotic	Use	Abbreviation	Product number
Amikacin	Axenic culture	AMK	A0365900 (Sigma Aldrich)
Anhydrotetracycline	Axenic culture	ATc	AC233135000 (Fisher Scientific)
Azithromycin	Mouse efficacy studies	AZM	NDC 42806-151-34 oral suspension (Epic Pharma)
Azithromycin-dihydrate	Axenic culture	AZM	PZ0007 (Sigma Aldrich)
Bedaquiline	Axenic culture	BDQ	A12327-5 (AdooQ Biosciences)
Capreomycin Sulfate	Axenic culture	CAP	PHR1716 (Sigma Aldrich)
Chloramphenicol	Axenic culture	CHL	C-105-5 (GoldBio)
Clarithromycin	Axenic culture	CLR	C9742 (Sigma Aldrich)
Clarithromycin	Mouse efficacy studies	CLR	NDC 0781-1962-60 tablets (Sandoz)
Clindamycin hydrochloride	Axenic culture	CLI	C-175-10 (GoldBio)
Clofazimine	Axenic culture	CLO	TCC2866 (VWR)
Ethambutol dihydrochloride	Axenic culture	EMB	E4630 (Sigma Aldrich)
Fusidic acid sodium salt	Axenic culture	FA	F0881 (Sigma Aldrich)
GSK3011724A	Axenic culture, macrophage infection	GSK'724A	NA
Isoniazid	Axenic culture, Mouse efficacy studies	INH	I3377 (Sigma Aldrich)
Kanamycin	Axenic culture	KAN	K-120-50 (GoldBio)
Levofloxacin	Axenic culture	LFX	28266 (Sigma Aldrich)
Lincomycin hydrochloride	Axenic culture	LNC	62143 (Sigma Aldrich)
Linezolid	Axenic culture	LZD	SML1290 (Sigma Aldrich)
Moxifloxacin	Axenic culture	MFX	Y0000703 (Sigma Aldrich)
Rifampicin	Axenic culture, macrophage infection	RIF	R0079 (TCI)
Rifampicin	Mouse efficacy studies	RIF	NDC 61748-018-30 tablets (Akorn)
Streptomycin sulfate	Axenic culture	STR	S-150-100 (GoldBio)
Sutezolid	Axenic culture	SZD	PZ0035 (Sigma Aldrich)
Telithromycin	Axenic culture	TLM	SML2162 (Sigma Aldrich)
Tigecycline hydrate	Axenic culture	TGC	PZ0021 (Sigma Aldrich)
Vancomycin hydrochloride	Axenic culture	VAN	V2002 (Sigma Aldrich)
Zeocin	Axenic culture	ZEO	J67140-8EQ (Alfa Aesar)
