# Supplementary material for: CRISPRi chemical genetics and comparative genomics identify genes mediating drug potency in Mycobacterium tuberculosis
Source: Nat Microbiol. 2022 May 30;7(6):766–79. doi: 10.1038/s41564-022-01130-y (PMC9159947; doi:10.1038/s41564-022-01130-y)
Supplement: Supplementary file 2 — Reporting Summary [file 41564_2022_1130_MOESM2_ESM.pdf]

nature portfolio

Corresponding author(s): Jeremy M. Rock

Last updated by author(s): Apr 11, 2022

Reporting Summary

Nature Portfolio wishes to improve the reproducibility of the work that we publish. This form provides structure for consistency and transparency in reporting. For further information on Nature Portfolio policies, see our Editorial Policies and the Editorial Policy Checklist.

Statistics

For all statistical analyses, confirm that the following items are present in the figure legend, table legend, main text, or Methods section.

n/a	Confirmed	
☐	☒	The exact sample size (*n*) for each experimental group/condition, given as a discrete number and unit of measurement
☐	☒	A statement on whether measurements were taken from distinct samples or whether the same sample was measured repeatedly
☐	☒	The statistical test(s) used AND whether they are one- or two-sided<br>*Only common tests should be described solely by name; describe more complex techniques in the Methods section.*
☒	☐	A description of all covariates tested
☒	☐	A description of any assumptions or corrections, such as tests of normality and adjustment for multiple comparisons
☐	☒	A full description of the statistical parameters including central tendency (e.g. means) or other basic estimates (e.g. regression coefficient) AND variation (e.g. standard deviation) or associated estimates of uncertainty (e.g. confidence intervals)
☐	☒	For null hypothesis testing, the test statistic (e.g. *F*, *t*, *r*) with confidence intervals, effect sizes, degrees of freedom and *P* value noted<br>*Give P values as exact values whenever suitable.*
☐	☒	For Bayesian analysis, information on the choice of priors and Markov chain Monte Carlo settings
☐	☒	For hierarchical and complex designs, identification of the appropriate level for tests and full reporting of outcomes
☐	☒	Estimates of effect sizes (e.g. Cohen's *d*, Pearson's *r*), indicating how they were calculated

Our web collection on statistics for biologists contains articles on many of the points above.

Software and code

Policy information about availability of computer code

Data collection	Data collection was done with custom scripts dependent on third-party tools
Data analysis	- GraphPad Prism (version 9.1.2)<br>- Microsoft Excel(365)<br>- MAGeCK analysis method (version 0.5.9.2) in python (version 2.7.16)<br>- subread-align (version 1.6.0)<br>- Sci Py package (version 1.2.2)<br>- FastTree (version 2.1.11 SSE3)<br>- Mykrobe(Version 0.9.0)<br>- GenomicAlignments (version 1.22.1)<br>- DESeq2 (version 1.30.1)<br>- Snippy (version 3.2-dev)<br>- QualiMap with the default parameters (version 2.2.2-dev)<br>- SpoTyping (version 2.1)<br>- iTol (https://itol.embl.de/)<br>- Rsamtools (Version 2.2.3)<br>- GenomicAlignments (Version 1.22.1)<br>- GenomicFeatures (Version 1.38.2)<br>- seqinr (Version 3.6-1)<br>- ggplot2 (Version 3.3.3)<br>- tidyverse (Version 1.3.1)<br>- genefilter (Version 1.68.0)<br>- Rsubread (Version 2.0.1)

- Biostrings (Version 2.54.0)
- SummarizedExperiment (Version 1.16.1)
- BiocParallel (Version 1.20.1)
- pandas (Version 1.4.1)
- ast (Version 3.10.4)
- readr (Version 1.4.0)
- reshape2 (Version 1.4.4)
- gplots (Version 3.1.1)
- dendextend (Version 1.14.0)
- RColorBrewer (Version 1.1-2)
- openpyxl (Version 3.0.5)
- circlize (Version 0.4.12)

For manuscripts utilizing custom algorithms or software that are central to the research but not yet described in published literature, software must be made available to editors and reviewers. We strongly encourage code deposition in a community repository (e.g. GitHub). See the Nature Portfolio guidelines for submitting code & software for further information.

Data

Policy information about availability of data

All manuscripts must include a data availability statement. This statement should provide the following information, where applicable:

- Accession codes, unique identifiers, or web links for publicly available datasets
- A description of any restrictions on data availability
- For clinical datasets or third party data, please ensure that the statement adheres to our policy

Raw sequencing data has been deposited to the Short Read Archive (SRA) under project number PRJNA738381. All screen results are available in Supplemental Data 1 and at pebble.rockefeller.edu. The custom code is available at https://github.com/rock-lab/CGI_nature_micro_2022.
H37Rv reference genome (NC_018143) was applied for alignment and SNPs calling. The accession numbers for previously sequenced strains used in Fig 5f, 6f, 8b are available in Source Data 4.

Field-specific reporting

Please select the one below that is the best fit for your research. If you are not sure, read the appropriate sections before making your selection.

☒ Life sciences ☐ Behavioural & social sciences ☐ Ecological, evolutionary & environmental sciences

For a reference copy of the document with all sections, see nature.com/documents/nr-reporting-summary-flat.pdf

Life sciences study design

All studies must disclose on these points even when the disclosure is negative.

Sample size	The growth and drug dose-responsive curves presented in the manuscript consist of at least three technical replicates and two independent experiments. We used three biologically independent replicates for in vitro killing assays (Fig 2c, Extended Data Figure 4g, Extended Data Figure 5b). Mouse experiments included at least 5 mice per group which was sufficient to identify differences in bacterial burden between groups in previously published experiments. All graphs show mice as individual data points.
Data exclusions	Extended Data Figure 1c and 1h: Due to sample loss, in EDF 1c the EMB "Med" day 1 sequencing results reflect two replicates, one of which was sequenced in technical duplicate (to have 3 replicates total). In EDF 1h, STR "Med" samples harvested for deep sequencing only reflect the day 1 and day 5 timepoints because the 10 day samples was lost during sample preparation and DNA extraction.
Replication	We have indicated the number of times experiment was independently performed as described in the figure legends and their corresponding methods.
Randomization	There was no randomization in this study because there were no features for which randomization was deemed necessary. Mice used in this study are female BALB/c mice (6-8 weeks of age), which are inbred and should be genetically identical to each other.
Blinding	Blinding was not performed for any of the in vitro or in vivo experimentation. The measurements of optical density, CFU, RNA expression do not require researcher-based judgments and therefore we deemed blinding not necessary

Reporting for specific materials, systems and methods

We require information from authors about some types of materials, experimental systems and methods used in many studies. Here, indicate whether each material, system or method listed is relevant to your study. If you are not sure if a list item applies to your research, read the appropriate section before selecting a response.

Materials & experimental systems

n/a	Involved in the study	
☒	☐	Antibodies
☒	☐	Eukaryotic cell lines
☒	☐	Palaeontology and archaeology
☐	☒	Animals and other organisms
☒	☐	Human research participants
☒	☐	Clinical data
☒	☐	Dual use research of concern

Methods

n/a	Involved in the study	
☒	☐	ChIP-seq
☒	☐	Flow cytometry
☒	☐	MRI-based neuroimaging

Animals and other organisms

Policy information about studies involving animals; ARRIVE guidelines recommended for reporting animal research

Laboratory animals	Bone marrow-derived macrophages (BMDMs) were differentiated from wild-type, female C57BL/6NTAC mice (Taconic Farms, 6-8 weeks of age). 7-8 weeks-old Female BALB/c mice (Charles Rivers Laboratory) were infected with approximately 100-200 CFU of Mtb using a whole-body inhalation exposure system (Glas-Col) at the Biosafety Level-3(BSL-3) Laboratory. Mice were kept at 12h light/12h dark cycle at an ambient temperature of 72 degrees +/-2 and a relative humidity of 40-60%
Wild animals	The study did not involve wild animals.
Field-collected samples	The study did not involve samples collected from the field.
Ethics oversight	All animal work was performed in accordance with the Guide for the Care and Use of Laboratory Animals of the National Institutes of Health, with approval from the Institutional Animal Care and Use Committee of Rockefeller University and Committee of the Center for Discovery and Innovation.

Note that full information on the approval of the study protocol must also be provided in the manuscript.
